# Supplementary material for: Identifying clinico-radiological determinants of post-stroke fatigue 3 months post-stroke in a French hospital-based cohort of non-severe stroke patients without psychiatric comorbidities
Source: PLoS One. 2026 Mar 23;21(3):e0345376. doi: 10.1371/journal.pone.0345376 (PMC13008045; doi:10.1371/journal.pone.0345376)
Supplement: S1 Table — Spearman’s correlations (ρ and p-value) between clinical factors to determine confounds for multivariate analyses. HAD-A: Hospital Anxiety and Depression–Anxiety score, HAD-D: Hospital Anxiety and Depression–Depression score, NIHSS: National Institute of Health Stroke Scale, MoCA: Montreal Cognitive Assessment. Bold: Significant results at p < 0.05. (DOCX) [file pone.0345376.s001.docx]

|  | | NIHSS (*ρ*;p) | | MoCA (*ρ*;p) | | HAD-A (*ρ*;p) | | HAD-D (*ρ*;p) | |
| --- | --- | --- | --- | --- | --- | --- | --- | --- | --- |
|  |  | T1 | T2 | T1 | T2 | T1 | T2 | T1 | T2 |
| Age |  | 0.057; 0.380 | -0.026; 0.692 | -0.316; **<0.001** | -0.288; **<0.001** | -0.040; 0.544 | -0.172; **0.009** | 0.125; 0.052 | 0.019; 0.773 |
| NIHSS | T1 |  | 0.495; **<0.001** | -0.038; 0.559 | 0.000; 0.997 | -0.044; 0.502 | -0.045; 0.497 | 0.054; 0.407 | 0.035; 0.592 |
|  | T2 |  |  | 0.068; 0.299 | -0.032; 0.625 | -0.027; 0.686 | -0.015; 0.825 | 0.047; 0.472 | 0.036; 0.590 |
| MoCA | T1 |  |  |  | 0.411; **<0.001** | 0.063; 0.338 | -0.066; 0.317 | -0.068; 0.290 | -0.146; **0.027** |
|  | T2 |  |  |  |  | -0.010; 0.885 | -0.044; 0.506 | -0.006; 0.924 | -0.128; 0.052 |
| HAD-A | T1 |  |  |  |  |  | 0.485; **<0.001** | 0.255; **<0.001** | 0.170; **0.011** |
|  | T2 |  |  |  |  |  |  | 0.171; **0.009** | 0.540; **<0.001** |
| HAD-D | T1 |  |  |  |  |  |  |  | 0.423; **<0.001** |
