# Supplementary material for: Identifying clinico-radiological determinants of post-stroke fatigue 3 months post-stroke in a French hospital-based cohort of non-severe stroke patients without psychiatric comorbidities
Source: PLoS One. 2026 Mar 23;21(3):e0345376. doi: 10.1371/journal.pone.0345376 (PMC13008045; doi:10.1371/journal.pone.0345376)
Supplement: S2 Table — The 14 items of the Hospital Anxiety and Depression (HAD) scale and the 20 items of the Mental Fatigue Inventory (MFI) are entered into an exploratory factor analysis using promax rotation and minimum residual extraction. The superscripts note the domain of the item from the two scales: HAD: AAnxiety and DDepression; MFI: AReduced Activity, GGeneral Fatigue, MEMental Fatigue, MOReduced Motivation, and PPhysical Fatigue. (DOCX) [file pone.0345376.s002.docx]

|  | **Factor** | | | | | |  |
| --- | --- | --- | --- | --- | --- | --- | --- |
|  | **1** | **2** | **3** | **4** | **5** | **6** | **Uniqueness** |
| **HAD(1)_A_** |  |  |  |  |  | 0.406 | 0.518 |
| **HAD(2)_D_** |  |  | 0.609 |  |  |  | 0.392 |
| **HAD(3)_A_** |  |  |  | 0.988 |  |  | 0.324 |
| **HAD(4)_D_** |  |  | 0.489 |  |  |  | 0.414 |
| **HAD(5)_A_** |  |  |  | 0.644 |  |  | 0.379 |
| **HAD(6)_D_** |  |  |  |  |  | 0.307 | 0.632 |
| **HAD(7)_A_** |  |  |  |  |  | 0.539 | 0.696 |
| **HAD(8)_D_** | 0.391 |  |  |  |  |  | 0.475 |
| **HAD(9)_A_** |  |  |  | 0.809 |  |  | 0.416 |
| **HAD(10)_D_** |  |  | 0.202 |  |  |  | 0.664 |
| **HAD(11)_A_** |  |  |  |  |  | 0.431 | 0.859 |
| **HAD(12)_D_** |  |  | 0.355 |  |  |  | 0.369 |
| **HAD(13)_A_** |  |  |  | 0.792 |  |  | 0.486 |
| **HAD(14)_D_** |  |  |  |  |  | 0.376 | 0.714 |
| **MFI(1)_G_** | -0.472 | 0.409 |  |  |  |  | 0.364 |
| **MFI(2)_P_** | 0.442 |  | 0.436 |  |  |  | 0.526 |
| **MFI(3)_ME_** |  | 0.740 |  |  |  |  | 0.301 |
| **MFI(4)_MO_** |  | 0.679 |  |  |  |  | 0.559 |
| **MFI(5)_G_** | 0.855 |  |  |  |  |  | 0.400 |
| **MFI(6)_A_** |  | 0.732 |  |  |  |  | 0.459 |
| **MFI(7)_ME_** |  | 0.330 |  |  | 0.644 |  | 0.304 |
| **MFI(8)_P_** | -0.476 | 0.502 |  |  |  |  | 0.403 |
| **MFI(9)_MO_** |  |  | 0.480 |  |  |  | 0.552 |
| **MFI(10)_A_** |  | -0.308 | 0.561 |  |  |  | 0.476 |
| **MFI(11)_ME_** |  |  |  |  | 0.657 |  | 0.262 |
| **MFI(12)_G_** | -0.617 |  |  |  |  |  | 0.304 |
| **MFI(13)_ME_** |  |  |  |  | -0.547 |  | 0.430 |
| **MFI(14)_P_** | 0.696 |  |  |  |  |  | 0.328 |
| **MFI(15)_MO_** |  | 0.660 |  |  |  |  | 0.551 |
| **MFI(16)_G_** | 0.853 |  |  |  |  |  | 0.360 |
| **MFI(17)_A_** |  |  | 0.532 |  |  |  | 0.674 |
| **MFI(18)_MO_** |  |  | 0.620 |  |  |  | 0.382 |
| **MFI(19)_ME_** |  |  | 0.395 |  | -0.541 |  | 0.423 |
| **MFI(20)_P_** | -0.726 |  |  |  |  |  | 0.290 |
