# Supplementary material for: Identifying clinico-radiological determinants of post-stroke fatigue 3 months post-stroke in a French hospital-based cohort of non-severe stroke patients without psychiatric comorbidities
Source: PLoS One. 2026 Mar 23;21(3):e0345376. doi: 10.1371/journal.pone.0345376 (PMC13008045; doi:10.1371/journal.pone.0345376)
Supplement: S3 Table — (DOCX) [file pone.0345376.s003.docx]

|  | **Factor 1** | **Factor 2** | **Factor 3** | **Factor 4** | **Factor 5** | **Factor 6** |
| --- | --- | --- | --- | --- | --- | --- |
| **Factor 1** | — | -0.614 | 0.540 | 0.539 | -0.533 | 0.296 |
| **Factor2** |  | — | -0.564 | -0.581 | 0.342 | -0.430 |
| **Factor 3** |  |  | — | 0.558 | -0.332 | 0.369 |
| **Factor4** |  |  |  | — | -0.353 | 0.575 |
| **Factor 5** |  |  |  |  | — | -0.211 |
| **Factor 6** |  |  |  |  |  | — |
