# Supplementary material for: Identifying clinico-radiological determinants of post-stroke fatigue 3 months post-stroke in a French hospital-based cohort of non-severe stroke patients without psychiatric comorbidities
Source: PLoS One. 2026 Mar 23;21(3):e0345376. doi: 10.1371/journal.pone.0345376 (PMC13008045; doi:10.1371/journal.pone.0345376)
Supplement: S4 Table — Univariate comparisons were done by either Mann-Whitney U tests with rank biserial correlation (rrb) as effect size or Chi-squared test of association (χ²); HAD-A: Hospital Anxiety and Depression scale – anxiety score, HAD-D: Hospital Anxiety and Depression scale – depression score, HSI: Heavy Smoking Index, MFI: Multidimensional Fatigue Inventory, MoCA: Montreal Cognitive Assessment, mRS: Modified Rankin Scale, NIHSS: National Institute of Health Stroke Scale. Bold: significant results at p < 0.05. (DOCX) [file pone.0345376.s004.docx]

|  | | **Men**  **mean (SD)** | **Men (%)** | **Women mean (SD)** | **Women (%)** | ***rrb* (p)** | **χ² (p)** |
| --- | --- | --- | --- | --- | --- | --- | --- |
| **Age** |  | 58.1 (14.1) |  | 55.0 (17.0) |  | 0.176 (0.09) |  |
| **Obesity** | T1 |  | 12.7 |  | 11.7 |  | 0.040  (1.000) |
| **Diabetes** | T1 |  | 12.9 |  | 5.9 |  | 2.437  (0.163) |
| **Hypertension** | T1 |  | 55.2 |  | 38.2 |  | 5.533 **(0.021)** |
| **Dyslipidemia** | T1 |  | 83.4 |  | 67.6 |  | 7.157 **(0.013)** |
| **Alcohol consumption** | T1 | 0.9 (1.4) | 67.9 | 0.2 (0.4) | 50.0 | 0.28 **(<0.001)** | 5.976  **(0.017)** |
|  | T2 | 0.8 (1.1) |  | 0.4 (0.8) |  | 0.13 (0.310) |  |
| **HSI** | T1 | 1.9 (1.6) | 28.2 | 1.4 (1.4) | 29.4 | 0.00 (0.433) | 0.033  (0.874) |
|  | T2 | 0.1 (0.4) |  | 0.0 (0.3) |  | 0.04 (0.632) |  |
| **mRS** | T2 | 0.7 (0.8) |  | 1.0 (0.8) |  | 0.02 **(0.003)** |  |
| **NIHSS** | T1 | 0.4 (0.8) |  | 0.5 (0.9) |  | 0.01 (0.938) |  |
|  | T2 | 0.2 (0.5) |  | 0.2 (0.5) |  | 0.03 (0.684) |  |
| **MoCA** | T1 | 26.1 (2.7) |  | 26.3 (2.8) |  | 0.04 (0.496) |  |
|  | T2 | 26.3 (2.7) |  | 25.1 (5.5) |  | 0.06 (0.376) |  |
| **HAD-A** | T1 | 5.8 (3.2) | 36.2 | 6.3 (2.7) | 48.5 | 0.10 (0.127) | 3.558 (0.073) |
|  | T2 | 4.9 (3.3) | 24.5 | 6.4 (3.6) | 42.6 | 0.20 (0.002) | 7.944 **(0.004)** |
| **HAD-D** | T1 | 2.4 (2.4) | 8.6 | 2.4 (2.2) | 4.4 | 0.01 (0.861) | 1.228 (0.206) |
|  | T2 | 3.2 (3.6) | 14.7 | 3.8 (3.2) | 17.6 | 0.13 **(0.047)** | 0.365 (0.337) |
| **MFI Total** | T2 | 43.8 (16.5) | 19.6 | 48.3 (15.5) | 23.5 | 0.10 **(0.036)** | 0.457 (0.593) |
| **MFI General** | T2 | 9.8 (4.3) | 32.5 | 11.8 (4.4) | 50.0 | 0.21 **(0.001)** | 6.372 **(0.016)** |
| **MFI Physical** | T2 | 9.2 (4.0) | 39.9 | 10.0 (4.3) | 45.6 | 0.08 (0.207) | 0.675 (0.462) |
| **MFI Reduced Motivation** | T2 | 8.0 (3.5) | 28.8 | 8.3 (3.1) | 36.8 | 0.08 (0.248) | 1.444 (0.274) |
| **MFI Reduced Activity** | T2 | 9.1 (3.5) | 33.7 | 9.7 (4.2) | 42.3 | 0.354 (0.06) | 1.692 (0.228) |
| **MFI Mental** | T2 | 7.7 (3.9) | 27.6 | 8.3 (3.6) | 27.9 | 0.112 (0.110) | 0.004 (1.000) |
| **Lesion Volume (mm3)** | T1 | 3218.5 (7489.8) |  | 3206.1 (5742.6) |  | 0.00 (0.971) |  |
| **Fazekas Periventricular (% ≥1)** | T1 |  | 55.4 |  | 45.5 |  | 1.848  (0.189) |
| **Fazekas**  **Deep (% ≥1)** | T1 |  | 47.5 |  | 47.1 |  | 0.006 (1.000) |
| **Brainstem Leukoencephalopathy** | T1 |  | 15.9 |  | 19.1 |  | 0.317 (0.568) |
| **Lobar Microbleeds (% >1)** | T1 |  | 9.8 |  | 5.9 |  | 0.826 (0.446) |
| **Deep Microbleeds (% >1)** | T1 |  | 10.4 |  | 7.4 |  | 0.435 (0.624) |
