# Supplementary material for: Identifying clinico-radiological determinants of post-stroke fatigue 3 months post-stroke in a French hospital-based cohort of non-severe stroke patients without psychiatric comorbidities
Source: PLoS One. 2026 Mar 23;21(3):e0345376. doi: 10.1371/journal.pone.0345376 (PMC13008045; doi:10.1371/journal.pone.0345376)
Supplement: S5 Table — Cells show F-statistic from Quade tests and p-value in parentheses. Bold: Significant results at p < 0.05. aadjusted for HAD Anxiety scores at T2. badjusted for HAD Depression scores at 3 months post-stroke. (DOCX) [file pone.0345376.s005.docx]

|  | F^a^(p) | F^b^(p) |
| --- | --- | --- |
| **MFI Total** | 0.369 (0.544) | 0.708 (0.401) |
| **General Fatigue** | 4.014 **(0.046)** | 6.389 **(0.012)** |
| **Physical Fatigue** | 0.022 (0.881) | 0.012 (0.911) |
| **Reduced Motivation** | 0.010 (0.920) | 0.021 (0.884) |
| **Reduced Activity** | 0.014 (0.907) | 0.087 (0.769) |
| **Mental**  **Fatigue** | 0.001 (0.979) | 0.361  (0.548) |
