# Supplementary material for: Identifying clinico-radiological determinants of post-stroke fatigue 3 months post-stroke in a French hospital-based cohort of non-severe stroke patients without psychiatric comorbidities
Source: PLoS One. 2026 Mar 23;21(3):e0345376. doi: 10.1371/journal.pone.0345376 (PMC13008045; doi:10.1371/journal.pone.0345376)
Supplement: S6 Table — Kruskal-Wallis tests between the three age groups: under 55, between 55–70 and above 70 years. HAD-A: Hospital Anxiety and Depression–Anxiety score, HAD-D: Hospital Anxiety and Depression–Depression score, MFI: Multidimensional Fatigue Inventory. Bold: Significant results at p < 0.05. (DOCX) [file pone.0345376.s006.docx]

|  | χ² | p |
| --- | --- | --- |
| General Fatigue | 0.81938 | 0.664 |
| Physical Fatigue | 1.95591 | 0.376 |
| Reduced Motivation | 0.38050 | 0.827 |
| Reduced Activity | 0.00879 | 0.996 |
| Mental Fatigue | **7.95135** | **0.019** |
| MFI Total | 0.43337 | 0.805 |
| HAD Anxiety T1 | 0.58017 | 0.748 |
| HAD Depression T1 | 4.02727 | 0.134 |
| HAD Anxiety T2 | 5.01895 | 0.081 |
| HAD Depression T2 | 0.86326 | 0.649 |
