# Supplementary material for: Identifying clinico-radiological determinants of post-stroke fatigue 3 months post-stroke in a French hospital-based cohort of non-severe stroke patients without psychiatric comorbidities
Source: PLoS One. 2026 Mar 23;21(3):e0345376. doi: 10.1371/journal.pone.0345376 (PMC13008045; doi:10.1371/journal.pone.0345376)
Supplement: Attachment — Submitted filename: Comments for Manuscript PONE.docx [file pone.0345376.s007.docx]

**Comments for Manuscript PONE-D-26-00845**

**Recommendation: Major Revision Required**

**1. Compromised Sample Representativeness**

The exclusion of patients with "any mood or anxiety disorder in the past 6 months" represents a stringent design choice. While this approach is justified from the perspective of reducing confounding factors, it makes the study cohort highly unrepresentative of the general post-stroke population (post-stroke depression and anxiety prevalence: 25-40%). Consequently, the prevalence of post-stroke fatigue (PSF) in this cohort (20.8%) is substantially lower than the reported population-level estimates (45.2%), which raises questions about the generalizability of the findings. More fundamentally, this exclusion strategy changes the research question from "what causes PSF?" to "what causes PSF in a specific subgroup of mild stroke patients without psychiatric comorbidities?"—a notably narrower inquiry.

Recommendation: The authors should more prominently highlight the specificity of their sample in both the abstract and the discussion. A sentence such as the following would enhance transparency: "This cohort is restricted to patients with mild-to-moderate ischemic stroke and no prior mood or anxiety disorders and may not be generalizable to patients with concurrent psychiatric conditions."

**2. Inconsistency with Existing Meta-Analytic Evidence**

A striking finding is that the authors report higher rates of general fatigue in female patients (M/W: 32.5%/50.7%, p=0.010), which directly contradicts a recent meta-analysis by Ozkan et al. (2024) concluding that female sex is not a significant PSF risk factor. However, the current discussion only briefly mentions "variability in sample characteristics" to dismiss this discrepancy, which is an insufficient explanation. The tension remains unresolved.

Recommendation: Conduct stratified subgroup analyses by sex and age groups (<55, 55-70, >70 years). Report the statistical significance of sex×age interaction terms (and other relevant interactions such as sex×HAD score).

**3. Conceptual Conflation of Fatigue and Depression Symptomatology**

Table 4 reveals a correlation of ρ=0.720 (p<0.001) between the Hospital Anxiety and Depression Scale-Depression (HAD-D) and the Multidimensional Fatigue Inventory (MFI) total fatigue score—a remarkably high association suggesting substantial symptom overlap. Although the authors correctly note in the introduction that fatigue and depression share overlapping experiential dimensions (e.g., energy deficit, psychomotor slowing; lines 61-65), the study does not statistically separate these constructs. The critical unresolved question is: What does this 0.72 correlation represent? Two independent but closely related symptom constructs? Different manifestations of a single underlying factor (measurement confounding)? A shared neurobiological mechanism? When the authors adjust for HAD scores (Table 6), the associations with MFI weaken but are not eliminated, suggesting potential measurement-level confounding rather than a true independent effect. This significantly undermines the interpretability of claims regarding the relative contributions of psychological versus neurobiological factors to PSF.

Recommendation: Conduct exploratory factor analysis or structural equation modeling to decompose MFI and HAD into common factors and unique factors. Reanalyze key associations using these decomposed factors rather than total scores. Explicitly discuss in the results and discussion the conceptual and statistical implications of symptom overlap, making it clear whether the reported associations reflect true independence or measurement-level confounding.

**4. PCA network analysis: insufficient specification of the multiple-testing family, FDR scope, and stability**

For the PCA-based network analysis, the manuscript does not adequately specify the testing family or the exact scope at which the false discovery rate (FDR) correction is applied. Given the modest effect sizes, transparency about the correction scope and component stability is essential.

Recommendation: Clearly define the family of tests subjected to FDR control (e.g., components×outcomes), report the variance explained and loading thresholds, and add a basic stability assessment (bootstrap or split-half). The Discussion should explicitly present these PCA results as exploratory/hypothesis-generating unless robust stability evidence is provided.
